# Malignant hypertension in a patient with Turner syndrome: A case report

**DOI:** 10.1097/MD.0000000000039128

**Published:** 2024-08-02

**Authors:** Ying Yang, Yong Ye, Huibo Wang, Hui Wu, Jing Zhang, Zhiyang Lv, Wen Li, Jian Yang

**Affiliations:** a Department of Cardiology, The First College of Clinical Medical Science, China Three Gorges University and Yichang Central People’s Hospital, Yichang, China; b Department of Cardiology, Institute of Cardiovascular Diseases, China Three Gorges University, Yichang, China; c Department of Cardiology, Hubei Key Laboratory of Ischemic Cardiovascular Disease, Yichang, China; d Department of Cardiology, Hubei Provincial Clinical Research Center for Ischemic Cardiovascular Disease, Yichang, China; e Department of Radiology, The First College of Clinical Medical Science, China Three Gorges University and Yichang Central People’s Hospital, Yichang, Hubei, China; f Department of Central Laboratory, Central Laboratory, The First College of Clinical Medical Science, China Three Gorges University and Yichang Central People’s Hospital, Yichang, China; g Department of Pediatrics, The First College of Clinical Medical Science, China Three Gorges University and Yichang Central People’s Hospital, Yichang, China.

**Keywords:** hypertension, malignant hypertension, primary reninism, Turner syndrome

## Abstract

**Rationale:**

Turner syndrome is characterized by complete or partial loss of the second sex chromosome. In patients with Turner syndrome, hypertension is well described. However, the literature regarding malignant hypertension is scarce. Therefore, an accurate and timely diagnosis and treatment are important.

**Patient concerns:**

A 13-year-old female with Turner syndrome presented to the emergency department with malignant hypertension, headache, spraying vomiting, convulsion, and loss of consciousness. Considering her medical history, symptoms, and auxiliary examination, secondary hypertension (primary reninism) was suspected, but without any occupying or hyperplasia in renal and adrenal.

**Diagnosis:**

A type of secondary hypertension, primary reninism.

**Interventions:**

The patient was immediately transferred to the pediatric intensive care unit. Subsequently, she was given nifedipine 0.35 mg/kg and captopril 0.35mg/kg to reduce blood pressure (BP), mannitol and furosemide to reduce cranial pressure, and phenobarbital and midazolam to terminate restlessness successively. Three hours later, the BP was consistently higher than 170/120 mm Hg, sodium nitroprusside was pumped intravenously, then, giving oral drug transition. Finally, she was given Valsartan-Amlodipine Tablets (I) (80 mg valsartan and 5 mg amlodipine per day) and bisoprolol (2.5 mg per day).

**Outcomes:**

For 2.5 years of follow-up, the BP reduced to 110–130/60–85 mm Hg, heart rate ranged between 65 and 80 bpm, and she could go to school without any headache, convulsion, and syncope.

**Lessons:**

The clinical phenotype of Turner syndrome is complex and varied, affecting multiple systems and organs. Turner syndrome with malignant hypertension is rare, so we should systematically evaluate secondary hypertension, target-organ damage, and accompanied by standard management when Turner syndrome presents with hypertension.

## 1. Introduction

Turner syndrome is characterized by complete or partial loss of the second sex chromosome, affecting approximately 1/4000~1/2000 live-born female infants.^[1]^ In patients with Turner syndrome, hypertension is well described.^[2]^ However, the literature regarding malignant hypertension is scarce. Additionally, patients with Turner syndrome develop hypertension at an early age and continue through adulthood, with mild-to-moderate elevation of blood pressure (BP) in essential hypertension and moderate-to-severe elevation of BP in secondary hypertension.^[3]^ We describe the first case of a girl with Turner syndrome and a type of secondary hypertension, primary reninism, presenting with malignant hypertension and hypertensive encephalopathy.

## 2. Case presentation

A 13-year-old female with Turner syndrome presented to the Emergency Department of Yichang Central People’s Hospital with headache, spraying vomiting, convulsion, and loss of consciousness. The patient was immediately transferred to the pediatric intensive care unit.

**Figure 1.**
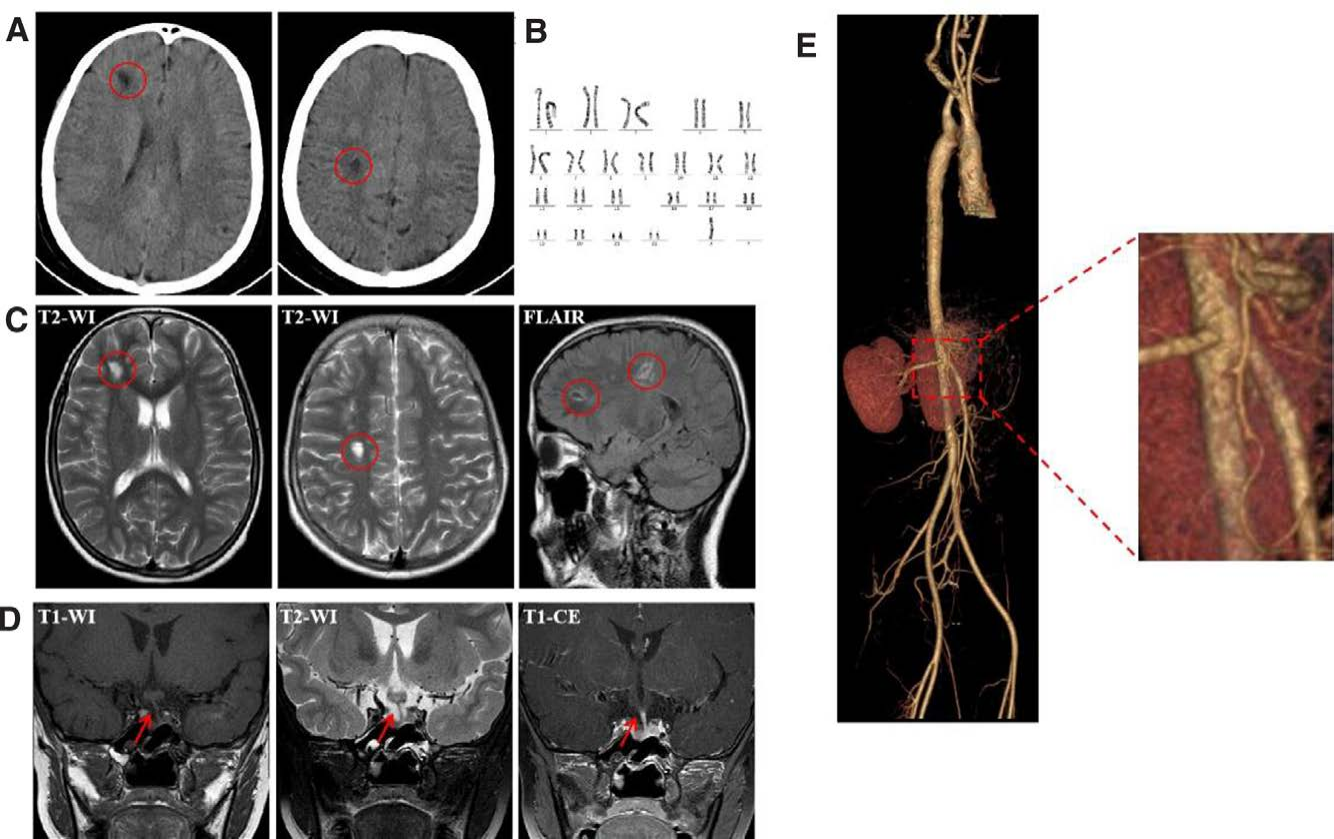
Imaging data and chromosome karyotype analysis. (A) Head CT, red circle marked low-density focus in the right frontal lobe and radiative crown; (B) chromosome karyotype analysis showed complete monosomy X (45, X); (C) brain MRI, red circle marked multiple abnormal signals in the right frontal lobe and radiative crown; (D) pituitary gland MRI, red arrow marked the pituitary stalk deviated to the left; (E) total aortic contrast-enhanced CT, the angle between the superior mesenteric artery and the abdominal aorta was 34.7°, without stenosis or occlusion. CT = computed tomography, MRI = magnetic resonance imaging.

Her head computed tomography (CT) showed low-density focus in the right frontal lobe and radiative crown (Fig. 1A). Additionally, her BP was severely elevated (190/140 mm Hg). Subsequently, she was given nifedipine 0.35 mg/kg and captopril 0.35 mg/kg to reduce BP, mannitol and furosemide to reduce cranial pressure, and phenobarbital and midazolam to terminate restlessness successively. Three hours later, the BP was consistently higher than 170/120 mm Hg (arm: right 170/120 mm Hg, left 175/120 mm Hg, leg: right 200/140 mm Hg, left 200/140 mm Hg). Furthermore, sodium nitroprusside was pumped intravenously, and the BP dropped to 150/100 mm Hg after 24 hours, then giving oral drug transition.

Two months ago, the patient was diagnosed with Turner syndrome with complete monosomy X (45, X) for short stature (height: 133.1 cm, weight: 29 kg, both <3 rd) and with no increase in height during the last 2 years. The chromosome karyotype analysis is shown in Figure 1B. As such, we did a series of tests to identify the reason for hypertension and convulsion. Blood tests showed normal blood routine and liver, kidney, and thyroid function. Electrolyte test showed potassium (K) 3.34 mmol/L↓, sodium (Na) 134.5 mmol/L↓, chloride (Cl) 91.2 mmol/L↓, carbon dioxide combining power (CO_2_CP) 29.9 mmol/L↑, urinary protein (PRO) +↑, and microalbuminuria 36.20 mg/L↑. Cerebrospinal fluid analysis was normal and without growth hormone deficiency in growth hormone stimulation tests. The catecholamines system was measured in the normal range (dopamine 0.089 nmol/L, adrenaline 0.66 nmol/L, norepinephrine 2.444 nmol/L, urine vanilla-bitter mandelic acid 10.79 mg/24 h). Moreover, the levels of serum sex hormone and cortisol at 8 am and 4 pm were normal, and adrenocorticotrophic hormone was 1.4 pg/mL↓. In contrast, the levels of the renin–angiotensin–aldosterone system (RAAS) were significantly increased (decubitus: renin 491.6 uIU/mL↑, aldosterone 956 pg/mL↑; orthostatic: renin > 500 uIU/mL↑, aldosterone > 1000 pg/mL↑). Brain magnetic resonance imaging showed multiple abnormal signals in the brain (Fig. 1C). A pituitary gland magnetic resonance imaging showed pituitary developmental variation (Fig. 1D). Electroencephalogram showed increased background slow wave activity. The electrocardiogram also showed sinus tachycardia at 182 bpm. Ultrasound showed no stenosis or occlusion in the arteries and veins of the extremities, renal, and carotid arteries. No structural and functional abnormalities were found on echocardiography. Chest and abdominal CT showed no abnormal lesions. Adrenal-enhanced CT showed no occupying or hyperplasia. Renal-enhanced CT showed no occupying and homogenous enhancement. Total aortic contrast-enhanced CT revealed that the angle between the superior mesenteric artery and the abdominal aorta was 34.7°, without stenosis or occlusion (Fig. 1E). Furthermore, whole exome sequencing of the family showed that the child was carrying a novel de novo heterozygous deletion Xp22.33-q28. Notably, after BP and heart rate (HR) reduced to normal, the levels of RAAS were still very high (decubitus and orthostatic: renin > 500 uIU/mL↑, aldosterone > 1000 pg/mL↑).

For 2.5 years of follow-up, she was given Valsartan-Amlodipine Tablets (I) (80 mg valsartan and 5 mg amlodipine per day) and bisoprolol (2.5 mg per day) for antihypertensive treatment. Following treatment, her BP reduced to 110–130/60–85 mm Hg, her HR ranged between 65 and 80 bpm, and she could go to school without headache, convulsion, and syncope. The patient and her parents have provided informed consent for the publication of the case, and this report is based on the CARE guidelines.

## 3. Discussion

Due to the variety of different definitions, evaluation of the prevalence of hypertension in children and adolescents is challenging.^[4]^ More rigorous studies have demonstrated that the overall prevalence of hypertension in childhood is 2–5% and the prevalence of elevated BP in the general pediatric population is about 13–18%.^[5]^ Regardless of the age of the children, it is recommended to carefully check for a secondary cause of hypertension.^[6]^

Though Turner syndrome is characterized by short stature and gonadal dysgenesis, the phenotypic manifestations are varied and encompass a wide variety of organ systems, including cardiovascular malformations and renal abnormalities.^[7]^ Studies have also reported that the prevalence of hypertension is between 21% and 40% in girls and adolescent females with Turner syndrome.^[8]^ Unfortunately, the etiology of hypertension in Turner syndrome is largely unknown.^[8]^ It is likely multifactorial, and recent hypotheses include altered sympathetic tone, vasculopathy, endocrine factors, renal disease, and congenital heart disease.^[8]^

Our case is the first to illustrate a girl with Turner syndrome and primary reninism. Notably, the pathogenesis of hypertension in patients with Turner syndrome is complex. For this case, we speculate that it may be related to hyperactivity of the sympathetic nervous system and the RAAS. Activation of the renal sympathetic nerve can also lead to vasoconstriction of the renal arteries, activation of the RAAS, and thus an increase in BP. The increase in HR and BP in patients with Turner syndrome may be related to increased excitability of the sympathetic nervous system and decreased sensitivity to catecholamines. Further stimulation of the sympathetic nervous system by orthostatic stimulation and the cold pressor test may lead to increased secretion of catecholamines and increased HR and BP in Turner syndrome.^[9]^ Moreover, sympathovagal balance or tone is reduced at night, and children with Turner syndrome have increased BP at night.^[10]^ Following renal denervation in a 6-year-old girl with Turner syndrome, her BP returned to normal 12 months after the operation.^[11]^ Therefore, reducing the excitability of the sympathetic nervous system in patients with Turner syndrome and inhibiting the activation of the RAAS may be potential targets for treating hypertension in patients with Turner syndrome.

Renin, also known as angiotensinogen, is a proteolytic enzyme released by the glomerular granule cells of the juxtaglomerular apparatus (also known as the juxtaglomerular complex), which can stimulate the synthesis and secretion of aldosterone by the adrenal cortex. Abnormal increases in renin are mainly caused by large secretions of renin by renal paraglomerular cell tumors, leading to severe diseases such as hypertension and hyperreninemia.^[12]^ In this case, the patient had no kidney or adrenal-related diseases, and the patient’s hypothalamic pituitary adrenal axis-related hormones were also within the normal range. However, the patient still had hyperreninemia, which is closely associated with malignant hypertension.^[13]^

In our case, we found a Turner syndrome patient with primary reninism. BP was controlled well with RAAS inhibitor (valsartan) and β blocker (bisoprolol) for a 2.5-year follow-up. However, we found no hyperplasia in renal and adrenal. Thus, we speculated that the significantly increased renin was secreted by abnormally activated juxtaglomerular cells, which could be verified by renal biopsy and pathological analysis, renin measurement bilaterally in the renal veins, and adrenal ^131^I-cholesterol scan. Unfortunately, such tests were not performed in our case, which is the important limitation of our study; we will identify the source of the abnormally elevated renin if consent was obtained from the patient and his parents.

In conclusion, the clinical phenotype of Turner syndrome is complex and varied, affecting multiple systems and organs. High risk of cardiovascular disease is the most serious health problem and the leading cause of death in patients with Turner syndrome. When Turner syndrome presents with hypertension, we should systematically evaluate secondary hypertension, target-organ damage, and accompanied by standard management.

## Acknowledgments

The authors thank AiMi Academic Services (www.aimieditor.com) for English language editing and review services.

## Author contributions

**Conceptualization:** Ying Yang, Yong Ye, Huibo Wang, Hui Wu, Jian Yang.

**Data curation:** Ying Yang, Jing Zhang, Wen Li.

**Funding acquisition:** Ying Yang, Jing Zhang, Huibo Wang, Jian Yang.

**Methodology:** Ying Yang, Yong Ye, Jing Zhang, Zhiyang Lv, Huibo Wang, Wen Li.

**Writing—original draft:** Ying Yang, Yong Ye.

**Writing—review & editing:** Ying Yang, Huibo Wang, Jian Yang.

**Software:** Yong Ye, Hui Wu.

**Project administration:** Jing Zhang, Zhiyang Lv, Huibo Wang, Hui Wu, Jian Yang.

**Validation:** Jing Zhang, Huibo Wang.

**Resources:** Zhiyang Lv, Huibo Wang, Wen Li.

**Visualization:** Zhiyang Lv, Hui Wu, Jian Yang.

**Formal analysis:** Wen Li.
